# Junctional ectopic tachycardia (JET)

**DOI:** 10.1002/joa3.12410

**Published:** 2020-07-27

**Authors:** Mohammad Alasti, Sam Mirzaee, Colin Machado, Stewart Healy, Logan Bittinger, David Adam, Emily Kotschet, Jack Krafchek, Jeffrey Alison

**Affiliations:** ^1^ Monash Cardiac Rhythm Management Department MonashHEART Monash Medical Centre Melbourne Vic. Australia

**Keywords:** arrhythmia, ectopic, focal, junctional tachycardia, pediatrics, postoperative

## Abstract

Junctional ectopic tachycardia (JET) is a tachyarrhythmia arising from the atrioventricular node and His bundle area. Enhanced normal automaticity has been postulated as the mechanism of JET in the majority of patients. It is more common in children and can be seen as congenital or in postoperative settings. It is often a narrow complex tachycardia but can present as a wide complex tachycardia as a result of aberrant conduction. Its differentiation from other arrhythmias especially atrioventricular nodal reentrant tachycardia (AVNRT) can be challenging. Medical treatment of JET is difficult, and catheter ablation remains the mainstay of treatment in refractory cases with a high risk of atrioventricular block and recurrence.

## Graphical abstract

Junctional ectopic tachycardia is often a narrow complex tachycardia due to enhanced normal automaticity and its differentiation from other arrhythmias especially AVNRT can be challenging. Since existing diagnostic tests have limitations to differentiate it from other arrhythmias, the diagnosis should be made based on both clinical and electrophysiologic findings.

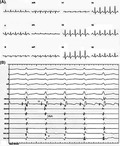

## 1 INTRODUCTION

Junctional Ectopic Tachycardia (JET) is a tachyarrhythmia arising from the atrioventricular node and His bundle area. It is also called junctional tachycardia, focal junctional tachycardia, or junctional nonreentrant tachycardia.

Heart rate in JET should be more than 95^th^ percentile of heart rate for age (typically more than 100 beats per minute in adults); otherwise, it is called accelerated junctional rhythm.^1^, ^2^, ^3^

JET is more common in children and may be congenital or acquired in postoperative settings. JET is a rare arrhythmia in adults and the pathogenesis is not completely understood. Moreover, because its clinical and electrocardiographic presentation varies, the diagnosis is challenging, and it can be easily mistaken for more common arrhythmias like atrioventricular nodal reentrant tachycardia (AVNRT). ^1^, ^4^, ^5^

## 2 CLINICAL FEATURES

This arrhythmia can be categorized as primary JET, without a clear predisposing factor, or secondary JET, which occurs in a clinical condition.

Primary JET occurs as congenital form or as sporadic cases in children and adults. Congenital JET occurs within the first 6 months of age, usually presents at birth, and is associated with high morbidity and mortality. It is an arrhythmia with mean ventricular rates of 200 to 250 beats/min and is associated with a high incidence of ventricular systolic dysfunction and clinical heart failure. Prenatal cases may present with hydrops.^1^

When it occurs after the age of 6 months, the clinical course is not malignant. Indeed, a similar clinical course to other supraventricular tachycardias has been reported in adults and the main symptom in these patients is palpitations with exacerbation during physical activity or stress. However, it may cause syncope or tachycardia‐mediated cardiomyopathy.^1^

Secondary JET is more common than primary JET, usually happening within 48 hours following surgical correction of congenital cardiac defects in children. Its prevalence has been reported 5%‐11%.^1^, ^4^

Postoperative JET occurs most commonly after surgery for abnormalities near to the atrioventricular junction, like Tetralogy of Fallot, atrioventricular septal defect, ventricular septal defect, and atrial septal defect. Although rare, JET can occur after any type of cardiac surgery, including Blalock‐Taussig shunt and extracardiac procedures.^1^, ^4^

Prolonged total surgical time, prolonged cardiopulmonary bypass time and aortic cross‐clamp time, age younger than 6 months, and postoperative administration of inotropic agents (such as milrinone, dopamine, nitric oxide, and vasopressin) have been considered as risk factors.^1^, ^4^, ^6^ It is also considered as a sign of atrioventricular (AV) node recovery when it occurs after postoperative AV block.^5^ Although often spontaneously resolves within a week, JET may cause significant hemodynamic compromise related to the loss of atrioventricular synchrony and rapid ventricular rates. Patients complicated with JET after surgery may have a longer stay in an intensive care unit, a higher incidence of need for mechanical ventilation and hemodynamic support, and a higher mortality rate.^1^, ^4^

Although postoperative JET is not common in adults, it can occur as a result of myocardial ischemia, metabolic, or autonomic disturbance or drug toxicity (eg, digoxin toxicity).^1^, ^6^

JET can also occur after ablation of AVNRT, and thus must be considered as a differential diagnosis for palpitations post‐AVNRT ablation, along with recurrence of AVNRT, inappropriate sinus tachycardia, and atrial fibrillation. Moreover, JET may occur during isoproterenol infusion during the electrophysiology (EP) study and should not be mistaken for re‐induction of AVNRT after ablation.^7^

## 3 PATHOPHYSIOLOGY

Although enhanced normal automaticity has been postulated as the mechanism of JET in the large body of evidence, the precise mechanism is not well understood. Some evidence suggests this arrhythmia may have various mechanisms, such as abnormal automaticity and triggered activity, in different clinical settings.^4^, ^5^, ^8^

Case reports have variously described JET as responsive to adenosine, overdrive pacing, and programmed stimulation, findings more consistent with triggered activity and abnormal automaticity than enhanced normal automaticity. In contrast to enhanced automaticity JET which shows transient suppression in response to overdrive pacing and adenosine, abnormal automaticity JET usually is not sensitive to those. Furthermore, arrhythmias as a result of an enhanced automaticity mechanism are not sensitive to calcium blockers, whereas arrhythmias with abnormal automaticity mechanism are. Conversely, JET with triggered activity is inducible by programmed stimulation and sensitive to adenosine too.^5^, ^8^

Autoimmune and genetic causes have been described for congenital JET. Positive family history can be found in almost half of patients and is associated with maternal lupus anti‐SSA and anti‐SSB in some.^9^, ^10^

In the postoperative condition, higher postoperative levels of troponin and CK‐MB correlate with the development of this arrhythmia. It has been postulated that postoperative JET may be a consequence of mechanical trauma or stretch with hemorrhage at the AV junction as a result of the disruption of ionic integrity of cell membranes. Increased sympathetic tone after surgery and ischemia with reperfusion injury of the AV node during longer cardiopulmonary bypass are other potential contributing factors.^6^, ^7^

JET is also seen in myocarditis and Lyme disease (which has a predilection for AV node area). Moreover, JET may be a sign for the return of normal sinus rhythm after treatment of Lyme disease.^11^

## 4 DIAGNOSIS

JET is often a narrow complex tachycardia, but the QRS morphology depends on differing rates of impulse propagation within the tri‐fascicular conduction system. Therefore, the ECG presentation of JET can change depending on arrhythmia cycle length, ventriculo‐atrial (VA) conduction, and arrhythmia duration (incessant vs paroxysmal).^12^

**FIGURE 1:**
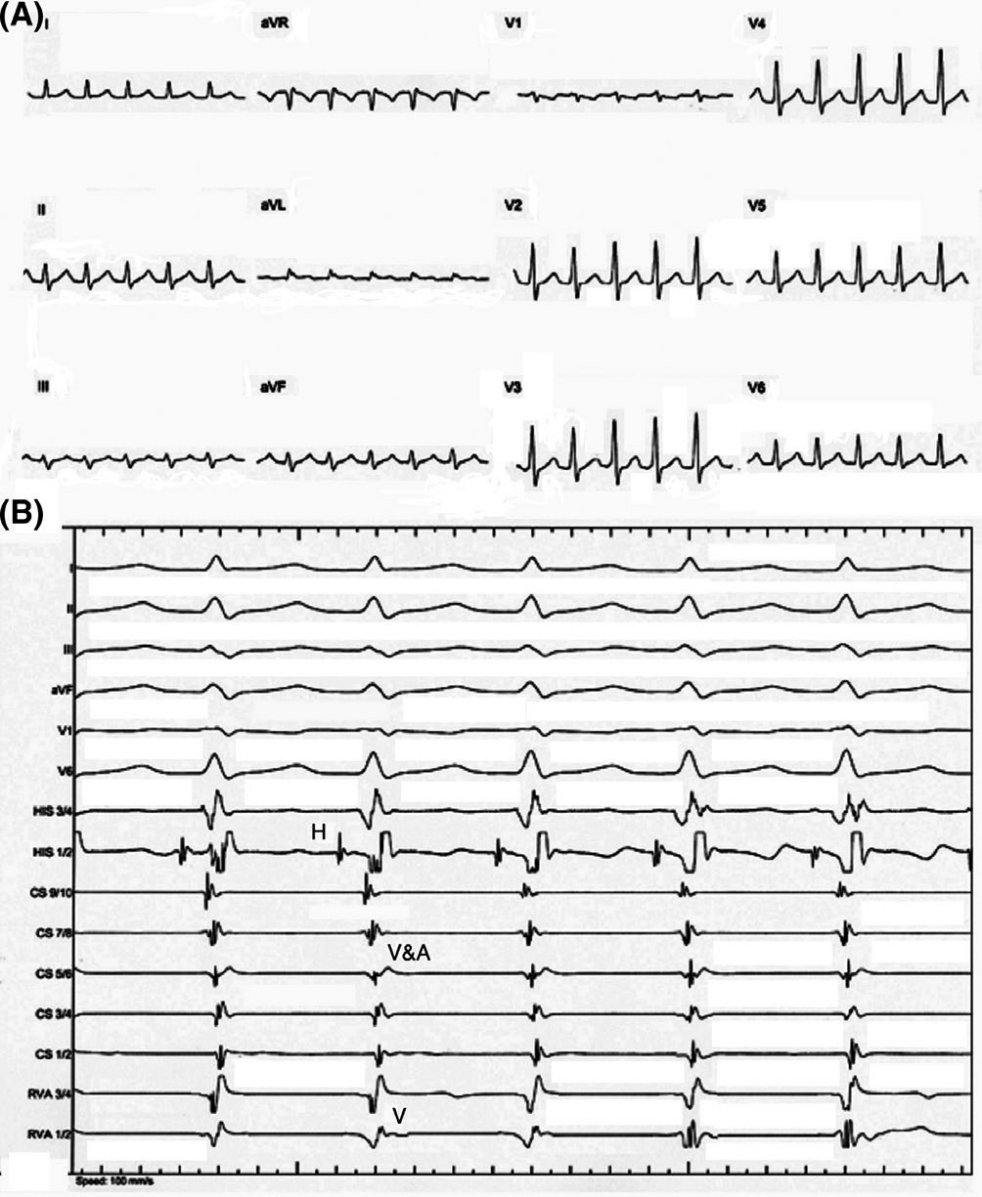
A, Twelve‐lead ECG showing a regular narrow complex tachycardia with a cycle length of 412 milliseconds and no obvious P waves before QRS complexes. B, Intracardiac recordings showing a tachycardia with one‐to‐one VA association and a cycle length of 412 milliseconds, HV interval of 45 milliseconds, and very short VA interval (A, atrial; H, His; V, ventricular)

The incessant form of JET with 1:1 VA conduction, is a regular, short RP, narrow complex tachycardia and similar to typical AVNRT (Figure 1A). To differentiate JET from AVNRT on surface ECG, the pattern of initiation of tachycardia can be helpful. Significant prolongation of PR interval seen on the initiating beat is suggestive of AVNRT, whereas the bursts of short RP tachycardia without initiation by an atrial premature beat (APB) or PR prolongation are in favor of JET. Moreover, the complete absence of VA conduction would suggest JET because AVNRT with entire upper common pathway block is less likely. A paroxysmal form of JET without VA conduction can present as an irregular narrow complex tachycardia caused by intermittent sinus capture beats.^1^, ^13^

JET with bundle branch block can mimic ventricular tachycardia arising from the conduction system, particularly if VA conduction is absent.^1^

JET can be diagnosed by observing VA dissociation during either ambulatory monitoring or during an EP study.^5^

## 5 DIFFERENTIAL DIAGNOSIS

The differential diagnosis of a narrow QRS tachycardia with AV dissociation includes AVNRT with upper common pathway block, JET, Orthodromic AV reentrant tachycardia using a concealed nodo‐fascicular or nodo‐ventricular accessory pathway, Intra‐Hisian reentrant tachycardia (very rare), and upper septal ventricular tachycardia (VT).^14^, ^15^

The differential diagnosis of a narrow QRS complex tachycardia with a very short VA interval includes AVNRT, JET, orthodromic AVRT using a concealed nodo‐fascicular or nodo‐ventricular accessory pathway, or, rarely, atrial tachycardia with prolonged PR interval.^15^, ^16^

## 6 DIFFERENTIATION DURING ELECTROPHYSIOLOGICAL STUDY

JET is a nonreentrant arrhythmia, so the lack of dependence on a critical coupling interval for tachycardia initiation, irregularity of the tachycardia, and inability to entrain are the main differentiating characteristics from a reentrant tachycardia.^5^

JET can typically be initiated by rapid atrial or ventricular pacing, or by isoprenaline infusion but not by programmed electrical stimulation, however, if the arrhythmia mechanism is triggered activity, it may be induced by programmed electrical stimulation.^17^

The hallmark finding during EP study is the presence of a His bundle electrogram preceding each QRS complex with a normal HV interval or even prolonged HV interval if there is infra‐Hisian conduction system disease. The presence of a short HV interval suggests a nodo‐fascicular accessory pathway, or an inter‐ or intrafascicular ventricular tachycardia.^1^

An A:V ratio of <1:1 is common during JET, occurring in up to 80% of cases.^15^ When VA conduction is present, differentiation of JET from atrial tachycardia is based on observation that H‐H interval variability drives A‐A interval variability.^1^

Differentiating JET from orthodromic AVRT using a nodo‐fascicular accessory pathway needs the introduction of a paced ventricular premature beat (VPB) during tachycardia when the His bundle is in the refractory period and observing the response of tachycardia. A His refractory VPB terminates AVRT or advances or delays the next His and resets the arrhythmia. His overdrive pacing is another useful technique for distinguishing AVRT from other arrhythmias. Entry into the tachycardia circuit and advancing the atrial electrogram within one beat is highly sensitive and specific for AVRT.^18^ Measurement of PPI‐TCL difference (postpacing interval minus tachycardia cycle length) during RV apical pacing may also differentiate these arrhythmias. Moreover, overdrive ventricular pacing of arrhythmia from basal RV septum can show a Post‐Pacing Interval (PPI) close to arrhythmia cycle length. It should be emphasized that although catheter‐induced ipsilateral bundle branch block can increase the HA interval and tachycardia cycle length, it may be minimal as the accessory pathway originates at septum near normal conducting system.^15^, ^19^, ^20^

Differentiating JET from AVNRT is crucial and challenging because catheter ablation of AVNRT is almost always successful with a very low incidence of AV block, whereas catheter ablation of JET has higher rates of AV block. During EP study, both arrhythmias have similar intracardiac electrogram activation patterns, similar HV intervals, and similar sites of earliest activation during catheter mapping (Figure 1B), and they may also be associated with VA block. Furthermore, JET may occur during isoproterenol infusion after slow pathway ablation and failure to diagnose of this arrhythmia may lead to further unnecessary ablation with an increased risk of AV block.^1^, ^2^, ^21^

Failure to entrain tachycardia favors a nonreentrant mechanism. Therefore, ventricular entrainment is particularly necessary during the EP study. The main issue is the entrainment of AVNRT is challenging to prove, as the antidromic wave front and orthodromic wave front from the preceding beat will collide in the AV node and there is no overt fusion during RV pacing, so the QRS morphology is identical to a paced beat.^22^ One useful maneuver is overdrive pacing with different cycle lengths during tachycardia. Overdrive pacing during a tachycardia with automaticity mechanism causes suppression of arrhythmia with an increase in return cycle length when pacing cycle length decreases, while overdrive pacing in an arrhythmia with reentrant mechanism entrains the arrhythmia with no significant change in return cycle length as pacing cycle length decreases.^23^

**FIGURE 2:**
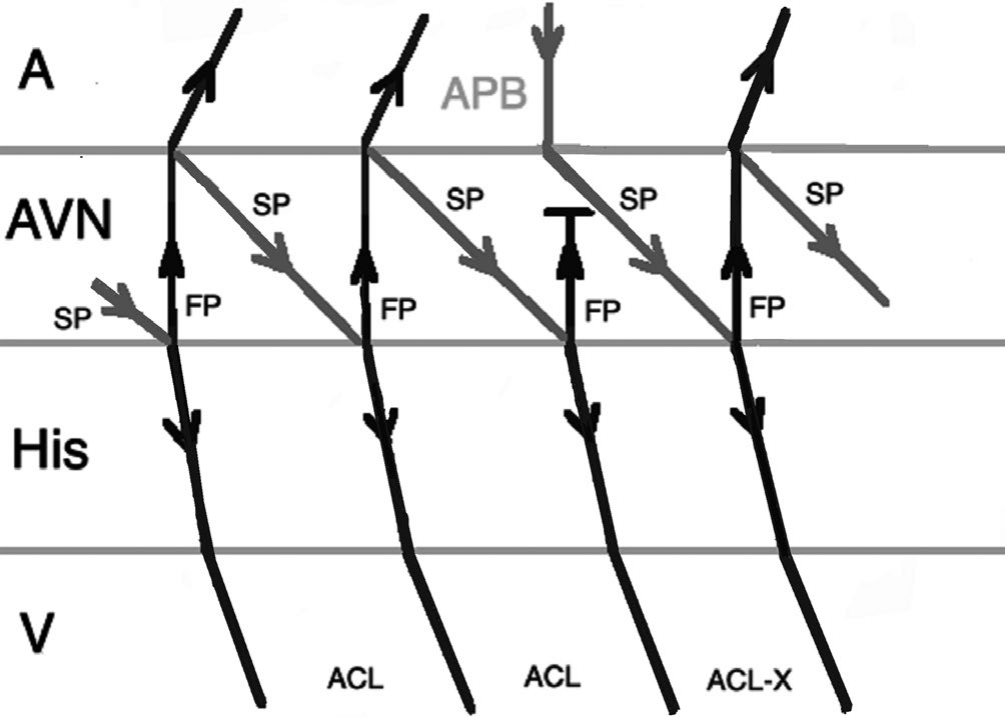
Response to an atrial premature beat delivered during His refractoriness (late APB) in AVNRT which does not influence the immediate beat but advances the next beat through conduction via slow pathway. (A, atrium; ACL, arrhythmia cycle length; APB, atrial premature beat; AVN, atrioventricular node; FP, fast pathway; SP, slow pathway; V, ventricle)

**FIGURE 3:**
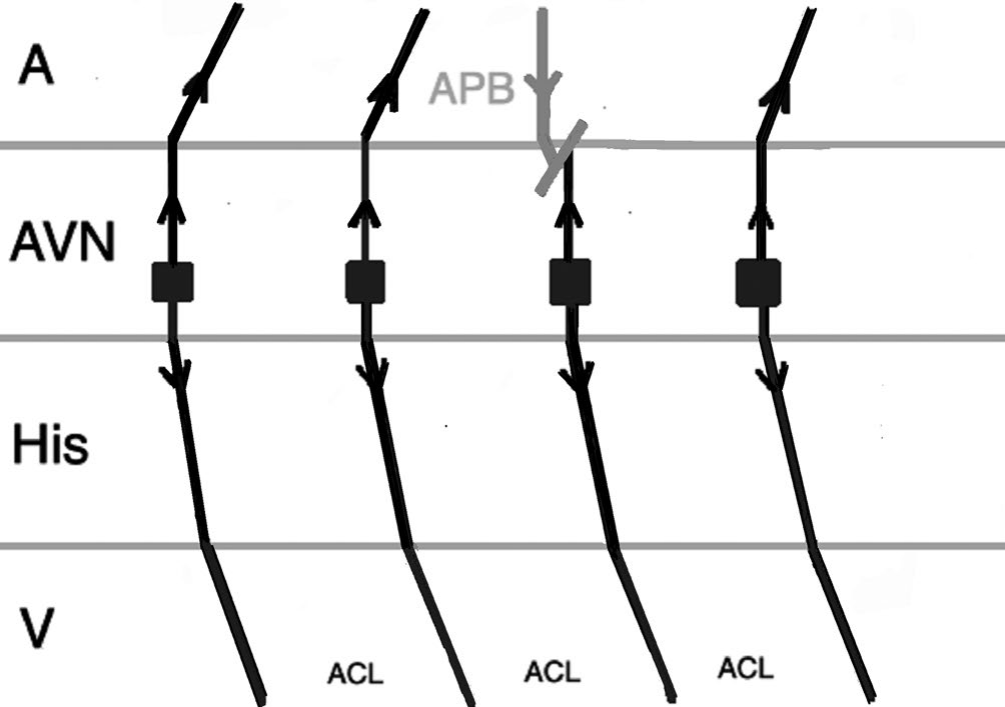
Response to a late APB in JET which does not influence the immediate and the next beats. (A, atrium; ACL, arrhythmia cycle length; APB, atrial premature beat; AVN, atrioventricular node; V, ventricle)

Another pacing maneuver to distinguish JET from AVNRT is the introduction of a paced Atrial Premature Beat (APB) during arrhythmia and observing the response. When a late‐coupled APB is delivered during His refractoriness (the local atrial activation in response to APB should occur simultaneously or after the His bundle activation on the His recording channel), any perturbation of the subsequent His with advancement or delay the subsequent ventricular beat favors the diagnosis of AVNRT. The APB can preexcite or delay the subsequent ventricular beat in AVNRT by engaging the anterograde slow AV nodal pathway (Figure 2). A late‐coupled APB delivered during His‐refractoriness does not affect JET (Figure 3).^1^, ^2^, ^24^

**FIGURE 4:**
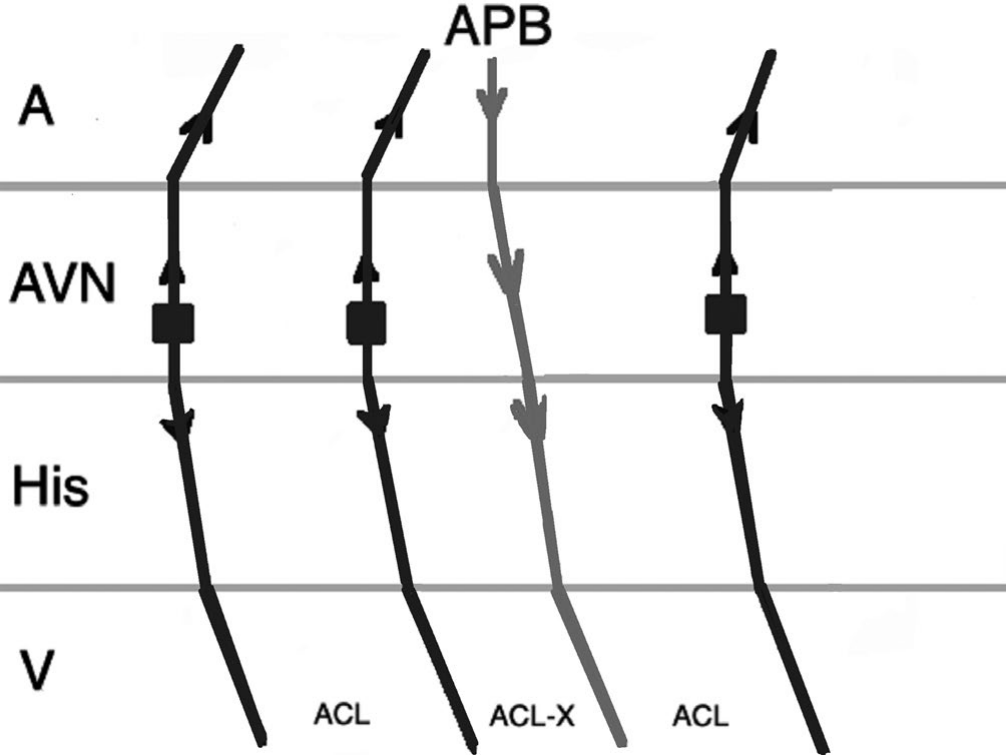
Response to an atrial premature beat delivered before His refractoriness (early APB) in JET which influences the immediate beat through conduction via fast pathway and the arrhythmia continues. (A, atrium; ACL, arrhythmia cycle length; APB, atrial premature beat; AVN, atrioventricular node; V, ventricle)

**FIGURE 5:**
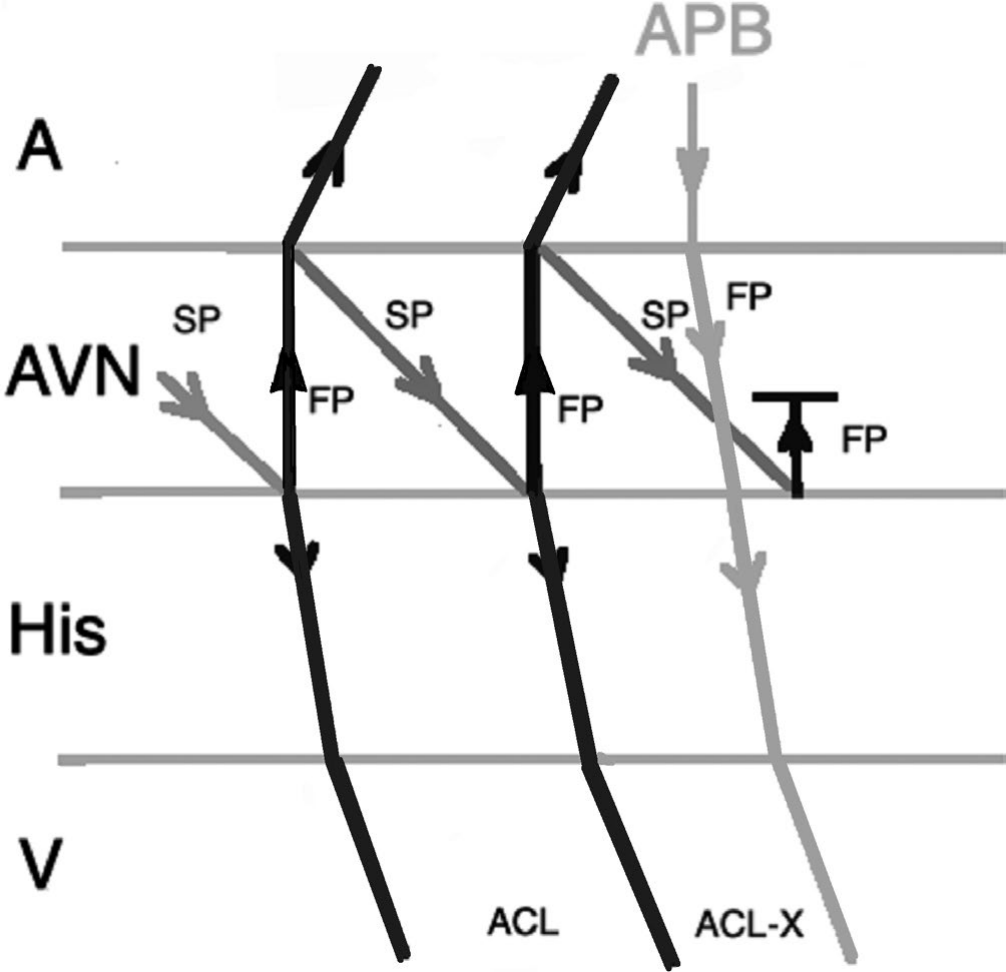
Response to an early APB in AVNRT which influences the immediate His through conduction via fast pathway and the arrhythmia terminates as a result of fast pathway refractoriness. (A, atrium; ACL, arrhythmia cycle length; APB, atrial premature beat; AVN, atrioventricular node; FP, fast pathway; SP, slow pathway; V, ventricle)

**FIGURE 6:**
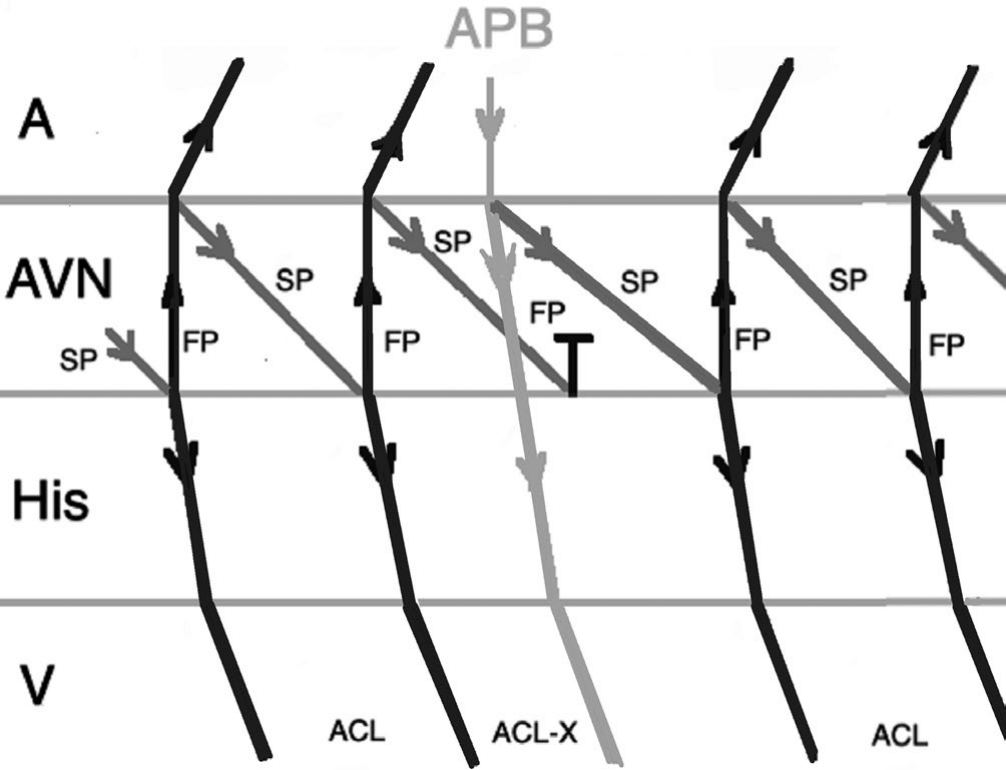
Response to an early APB in AVNRT with simultaneous anterograde fast and slow AV nodal pathways conduction. Therefore, AVNRT continues after an APB that advances the immediate His. (A, atrium; ACL, arrhythmia cycle length; APB, atrial premature beat; AVN, atrioventricular node; FP, fast pathway; SP, slow pathway; V, ventricle)

**FIGURE 7:**
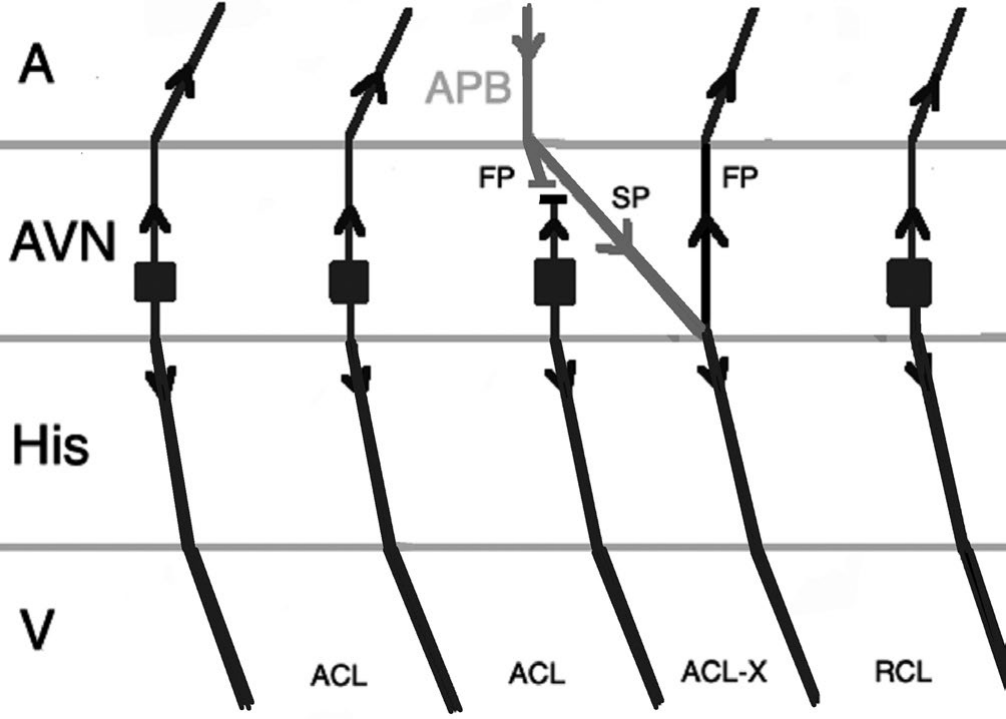
Response to an atrial premature beat delivered during His refractoriness in JET when dual AV node physiology is present. It can lead to misdiagnosis of JET as AVNRT. (A, atrium; ACL, arrhythmia cycle length; APB, atrial premature beat; AVN, atrioventricular node; FP, fast pathway; RCL, return cycle length; SP, slow pathway; V, ventricle)

On the other hand, an early‐coupled APB that captures the His may advance the immediate JET or AVNRT beat, but the tachycardia can only continue when there is a focal mechanism. Therefore, JET tends to sustain, in contrast to AVNRT which terminates as a result of refractory fast pathway as a result of antegrade conduction (Figures 4 and 5).^1^, ^24^ There are exemptions to this rule which may result in misdiagnosis of JET. When an early‐coupled APB captures the His and a double response to APB (simultaneous anterograde fast and slow AV nodal pathway conduction) occurs, it will advance the immediate His with switching the arrhythmia from slow‐fast to slow‐slow AVNRT thus continuation of the tachycardia (Figure 6). Moreover, if dual AV nodal physiology is present, introducing APBs during His refractoriness can lead to misdiagnosis of JET as AVNRT. Incidental anterograde slow pathway conduction can occur after the retrograde atrial activation from each JET beat. So, an APB timed to His refractoriness during JET can potentially advance (but not delay or terminate) the next tachycardia beat (Figure 7).^24^, ^25^

**FIGURE 8:**
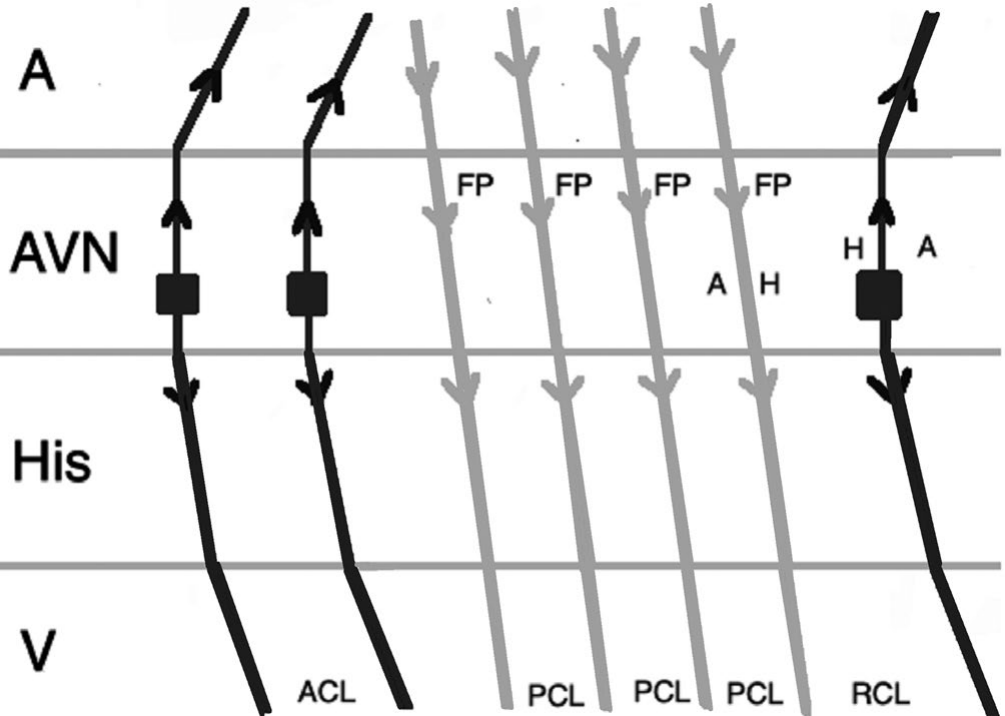
Response to atrial overdrive pacing in JET: after termination of pacing, the first return beat (arrhythmia) begins with an H followed by an A (A‐H‐H‐A response). (A, atrium; ACL, arrhythmia cycle length; AVN, atrioventricular node; FP, fast pathway; H, His; PCL, pacing cycle length; RCL, return cycle length; V, ventricle)

**FIGURE 9:**
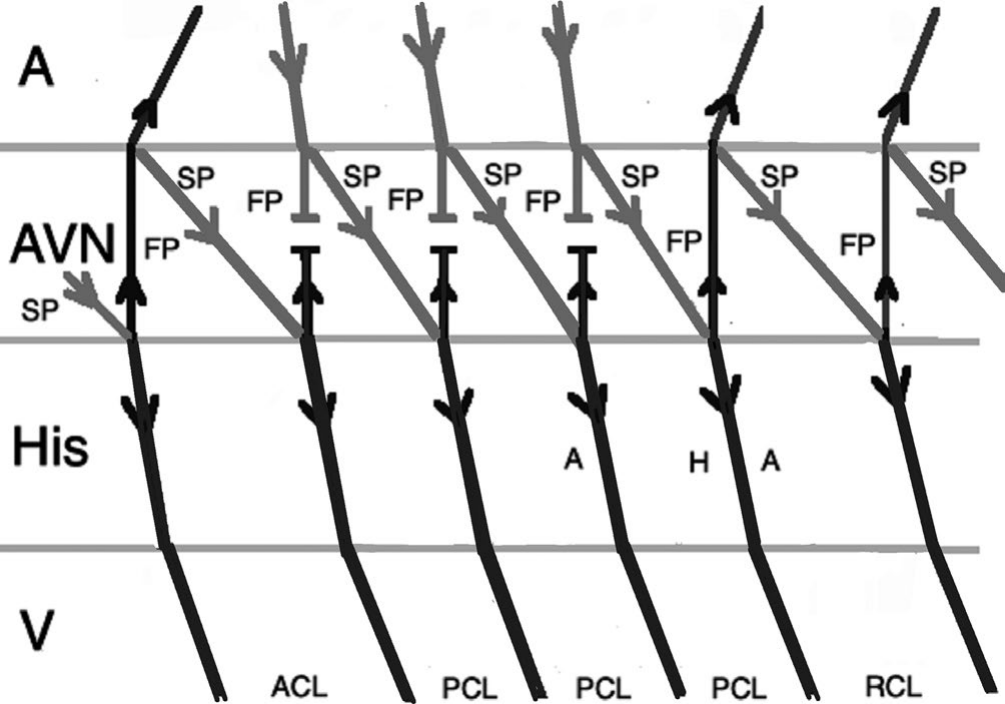
Response to atrial overdrive pacing in AVNRT: after termination of pacing, the first return beat begins with an A (A‐H‐A response). (A, atrium; ACL, arrhythmia cycle length; AVN, atrioventricular node; FP, fast pathway; H, His, PCL, pacing cycle length, RCL, return cycle length, SP, slow pathway, V, ventricle)

Atrial pacing at a rate slightly faster than the tachycardia during JET (overdrive pacing) will transiently suppress the arrhythmia after cessation of pacing and the tachycardia will resume with a junctional beat, beginning with a His signal. This will result in an Atrial‐His‐His‐Atrial (A‐H‐H‐A) response (Figure 8), while during atrial overdrive pacing of AVNRT, tachycardia will be entrained, and the anterograde AV nodal conduction uses only the slow pathway because the fast pathway is refractory from retrograde conduction. After cessation of pacing, tachycardia will resume after the last paced beat. The last atrial paced beat during typical AVNRT should conduct anterograde down the slow pathway and exit into the His‐Purkinje system while echoing up the fast pathway and exiting into the atrium. This would result in an Atrial‐His‐Atrial (A‐H‐A) response (Figure 9).^1^, ^25^

**FIGURE 10:**
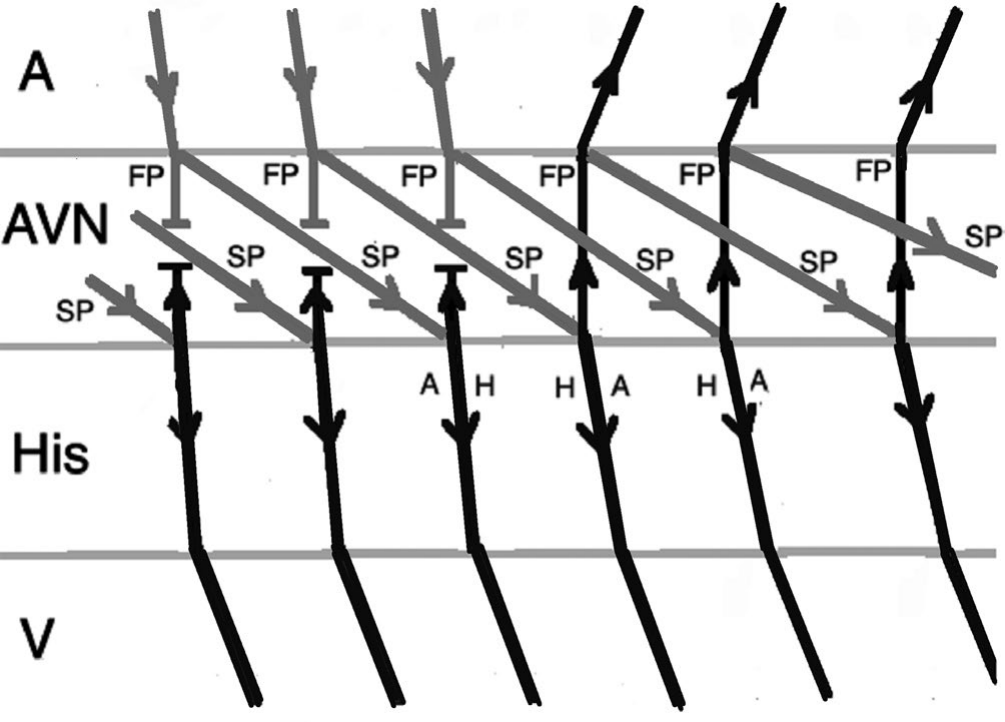
A pseudo A‐H‐H‐A response after overdrive pacing in AVNRT. The first beat after stopping pacing is not related to the last atrial paced beat as a result of slow conduction via slow pathway. (A, atrium; ACL, arrhythmia cycle length; AVN, atrioventricular node; FP, fast pathway; H, His; PCL, pacing cycle length; RCL, return cycle length; SP, slow pathway; V, ventricle)

It is imperative to correctly identify the last advanced His signal after cessation of pacing. After termination of pacing, often the immediate His signal is the result of the last atrial paced beat, but as a result of delayed conduction in the slow pathway, it may be related to the atrial paced beat before the cessation of pacing. This results in a pseudo A‐H‐H‐A response in AVNRT (Figure 10). The mechanism of this response is similar to pseudo Ventriculo‐Atrial‐Atrio‐Ventricular (V‐A‐A‐V) response that can happen during ventricular overdrive pacing in AVNRT. The best way to avoid this error is measuring H‐H intervals during and after atrial overdrive pacing. The last interval of the same cycle length as the pacing cycle length represents the His signal from the last atrial paced beat. If an atrial signal follows that His, an A‐H‐A response will be diagnosed.^25^

**FIGURE 11:**
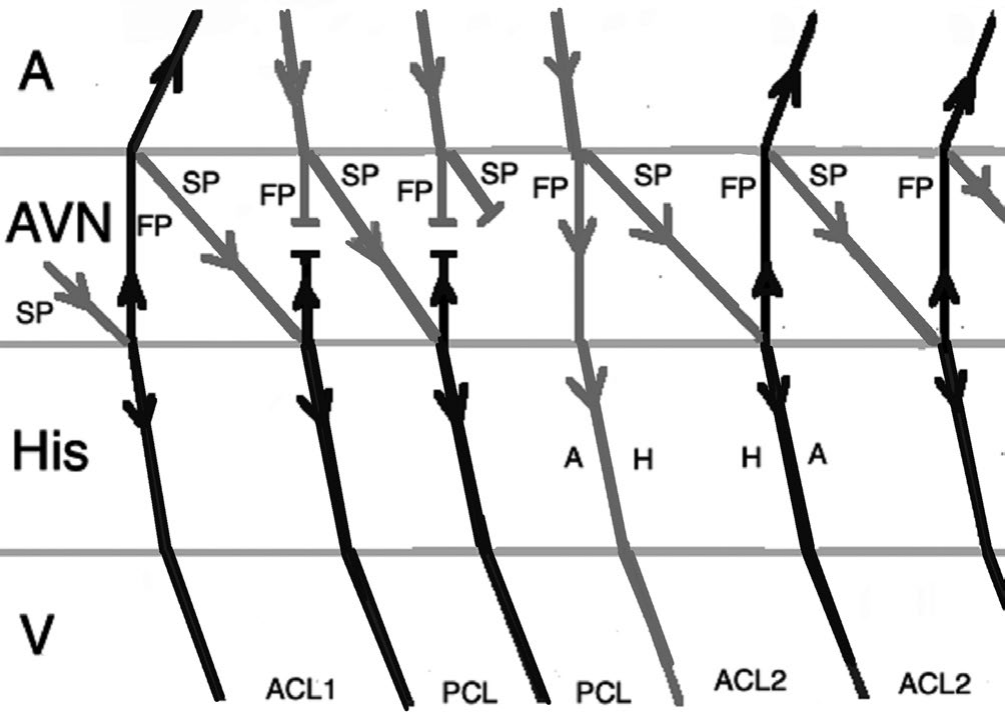
An A‐H‐H‐A response after overdrive pacing in AVNRT. During atrial pacing, the tachycardia (arrhythmia 1) is terminated and the last atrial paced beat results in two His signals as conduction proceeds anterograde down both the fast pathway and slow pathway and reinitiates the tachycardia (arrhythmia 2). (A, atrium; ACL_1_, arrhythmia 1 cycle length; ACL_2_, arrhythmia 2 cycle length; AVN, atrioventricular node; FP, fast pathway; H, His; PCL, pacing cycle length; SP, slow pathway; V, ventricle)

When a double ventricular response is present, AVNRT may be misdiagnosed as JET. Where AVNRT is ended during atrial overdrive pacing, the last atrial paced beat can result in two His signals as conduction proceeds anterograde down both the fast pathway and the slow pathway. If conduction down the slow pathway is able to echo up the fast pathway, reinitiating the tachycardia, an A‐H‐H‐A response would be observed, leading to the misdiagnosis of the rhythm as JET (Figure 11). This scenario should be anticipated when a double ventricular response is seen during baseline EP study. In addition, during atrial overdrive pacing, termination of the tachycardia by pacing can be recognized by an abrupt change in the AH interval because AV conduction switches from the slow pathway to the fast pathway.^25^

**FIGURE 12:**
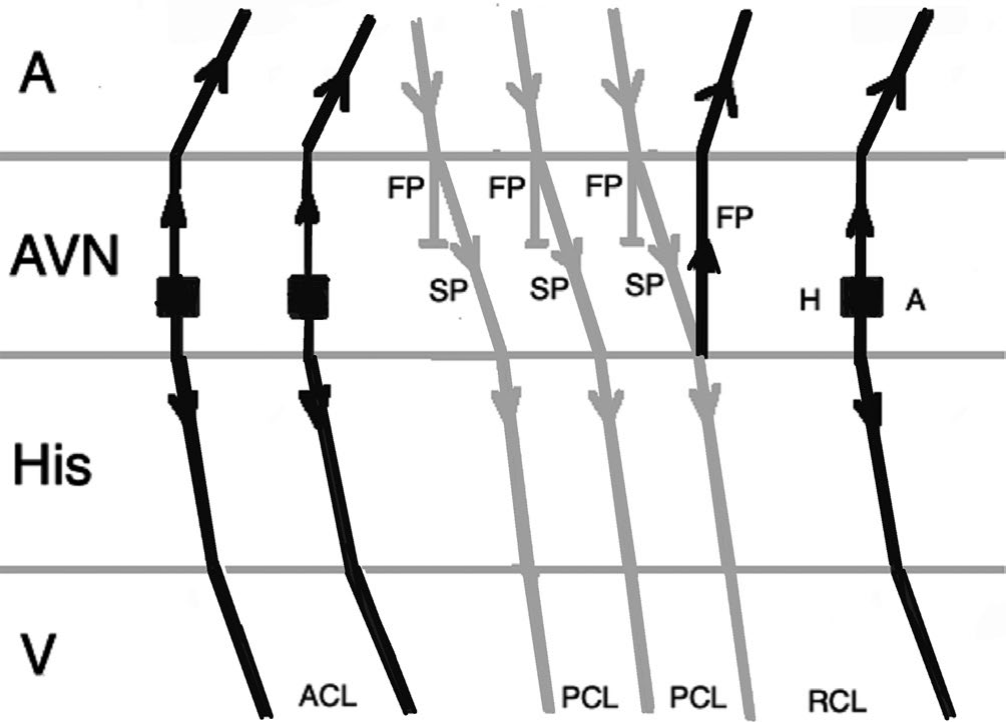
An A‐H‐A response after overdrive pacing in JET. When anterograde fast pathway conduction is poor, atrial overdrive pacing during JET conducts down the slow pathway and up the fast pathway causing an echo beat, results in an A‐H‐A response. (A, atrium; ACL, arrhythmia cycle length; AVN, atrioventricular node; FP, fast pathway; H, His; PCL, pacing cycle length; RCL, return cycle length; SP, slow pathway; V, ventricle)

Another possibility for JET to be misdiagnosed as AVNRT is when the anterograde fast pathway conduction is weak, seen during EP study (prolonged AH interval), or as a consequence of injury during ablation. In this condition, atrial overdrive pacing during JET can conduct down the slow pathway and up the fast pathway causing echo beat, resulting in an A‐H‐A response (Figure 12).^25^

Another pacing maneuver to distinguish JET from AVNRT is measuring the difference between HA interval during tachycardia and basal right ventricular pacing. The delta HA is calculated as the HA (pacing) minus the HA (tachycardia). A negative value indicates AVNRT and a positive value indicates JET. The relatively low specificity, difficulty in obtaining clear retrograde His recordings, and the dependency of this test to responses on the site of arrhythmia origin could limit the feasibility of this method in routine practice.^24^, ^25^

It has been suggested that the responses to appropriately timed APBs and atrial overdrive pacing can differentiate the two rhythms with high specificity and sensitivity. However, it should be mentioned that almost all of the studies had a relatively small sample size and majority of cases who were recruited in these trials had only spontaneous JET after ablation of AVNRT, not those with rare primary JET. Furthermore, postoperative JET and pediatric patients with congenital JET were not represented in these studies.^24^, ^25^ Since the existing diagnostic tests have limitations to differentiate JET from AVNRT, the diagnosis of JET remains challenging and should be made based on both clinical (including the clinical setting in which the arrhythmia has happened) and electrophysiologic findings.^2^, ^24^

## 7 MANAGEMENT

Treatment of JET is a challenging part of this rare arrhythmia. Clinical indications for treatment are symptoms, hemodynamic compromise, ventricular dysfunction, congestive heart failure, or evidence of hydrops in fetal cases.^1^ The congenital form of JET is often associated with higher morbidity and mortality rates, and it is not amenable to antiarrhythmic therapy.^5^ In patients with postoperative JET, interdisciplinary collaboration among surgeons, cardiologists, and intensive care physicians is necessary to manage this arrhythmia. Nearly all protocols in this setting use deep sedation, correction of electrolyte disturbances, rapid weaning of catecholaminergic agents, maintenance of mechanical ventilation, avoidance of fever, and surface cooling. These strategies can be therapeutic in a majority of cases. If the arrhythmia continues despite using the above recommendations, a combination of overdrive atrial pacing, medication, and hypothermia should be utilized. If symptoms continue to persist despite medication, ablation of the focus should be offered.^26^

## 8 PHARMACOLOGICAL THERAPIES

Recommended pharmacotherapy to suppress JET includes amiodarone, beta‐blockers, sotalol, flecainide, procainamide, digoxin, and anti‐inflammatory agents such as steroids or even colchicine.^7^, ^27^ Among these, amiodarone seems to be the most effective antiarrhythmic, when used as a single agent or in combination with others. Digoxin is not effective when used as monotherapy, but it is useful in combination with other antiarrhythmic agents, particularly in the setting of symptomatic heart failure.^1^ Emerging evidence indicates other antiarrhythmic agents such as ivabradine (an I_f_ blocker), nifekalant (a pure I_kr_ blocker), and landiolol (an ultra‐short acting super‐selective beta‐1 blocker) may have roles in suppressing JET.^28^, ^29^

Preoperative use of intravenous nitroprusside, the administration of magnesium after the rewarming phase of cardiopulmonary bypass, and preoperative use of propranolol have been demonstrated to diminish the incidence of JET. In addition, dexmedetomidine, an alpha‐2 adrenoreceptor agonist, administered pre‐ or intraoperatively, was shown to decrease the incidence and duration of JET.^6^, ^30^

## 9 ABLATION

Catheter ablation remains the mainstay of treatment in refractory JET unresponsive to medical therapy and it can be aided by using an electroanatomic mapping system.^31^ However, it is associated with a high risk of atrioventricular nodal block.^1^, ^2^, ^4^ When there is VA conduction, radiofrequency ablation can be done at the site of earliest retrograde atrial activation. When there is no VA conduction, it can be done anatomically in the posterior septum near the slow pathway location and if not effective, moving anteriorly. If a clear His electrogram cannot be seen, para‐Hisian pacing can help to find the location with the highest risk of AV block. A recent multicentre study has shown a very high rate of recurrence (53%) and high risk of AV block (21.4%) after catheter ablation of idiopathic JET in adults.^32^ In another study with a modest sample size study (n = 11), the rate of complete heart block was almost 1:5 (18%).^26^

Owing to the high risk of AV block, cryoablation is being used in many centers as the preferred method. Cryoablation should be performed at the focus where cryomapping suppresses the arrhythmia. Although the success rate in both radiofrequency ablation (RFA) and cryoablation has been reported approximately equal in a study included 44 pediatric patients (82% in RFA with recurrence rate of 13% (vs 85% in cryoablation with recurrence rate of 14%), the risk of complete heart block seems to be significantly lower in cryoablation.^3^ Interestingly, JET in children responds to catheter ablation better, with higher success and lower rates of AV block, suggesting that the involved cells are not intrinsic to AV junction.^1^

It has been claimed that the mapping of noncoronary cusp and empiric ablation at the site of earliest His signal on the cusp is safer than ablating close to the AV node in the right atrium.^32^

Finally, in the refractory symptomatic patients, AV junctional ablation and pacing remains the last resort, with accurate ablation of the junctional focus essential.^1^, ^3^

## 10 CONCLUSIONS

Junctional ectopic tachycardia is a rare arrhythmia and its diagnosis and management remain challenging. Since existing diagnostic tests have limitations to differentiate JET from other arrhythmias, the diagnosis of JET should be made based on both clinical and electrophysiologic findings.

## CONFLICT OF INTEREST

The authors declare no conflict of interests for this article.

## References

[1] Liu CF , Ip JE , Markowitz SM , Lerman BB . Junctional tachycardia In: Zipes D, Jalife J, Stevenson W, editors. Cardiac electrophysiology: from cell to bedside, 7th edn Philadelphia, PA: Elsevier, 2018; p. 768–775.

[2] Gonzalez MD , Banchs JE , Moukabary T , Rivera J . Ablation of atrioventricular junctional tachycardia: atrioventricular variants and focal junctional tachycardia In: Huang SKS, Miller JM, editors. Catheter ablation of cardiac arrhythmias, 4th edn Philadelphia, PA: Elsevier, 2020; p. 316–348.

[3] Collins KK , Van Hare GF , Kertesz NJ , Law IH , Bar‐Cohen Y , Dubin AM , Etheridge SP , Berul CI , Avari JN , Tuzcu V , Sreeram N , Schaffer MS , Fournier A , Sanatani S , Snyder CS , Smith RT , Arabia L , Hamilton R , Chun T , Liberman L , Kakavand B , Paul T , Tanel RE . Pediatric non post‐operative junctional ectopic tachycardia: Medical management and interventional therapies. J Am Coll Cardiol. 2009;53(8):690–7. 19232902 10.1016/j.jacc.2008.11.019

[4] Ozyilmaz EY , Ozyilmaz S , Guzeltas A . Junctional ectopic tachycardia in late period after early postoperative complete atrioventricular block: Messenger of return to normal sinus rhythm? Explanation with four case series. J Electrocardiol. 2017;50:378–382. 28189280 10.1016/j.jelectrocard.2017.01.015

[5] Pierick AR , Law IH , Muldonado JR , Bergen NH . Junctional ectopic tachycardia localization and procedural approach using cryoablation. Pacing Clin Electrophysiol. 2017;40:655–660. 28097671 10.1111/pace.13022

[6] Moak JP , Arias P , Kaltman JR , Cheng Y , Mccarter R , Hanumanthaiah S , Martin GR , Jonas RA . Postoperative junctional ectopic tachycardia: risk factors for occurrence in the modern surgical era. Pacing Clin Electrophysiol. 2013;36:1156–1168. 23662635 10.1111/pace.12163

[7] Kusterer N , Morales G , Butt M , Darrat Y , Parrott K , Ogunbayo G , Bidwell K , Patel R , Delisle B , Czarapata M , Elayi CS . Junctional ectopic rhythm after AVNRT ablation: an underrecognized complication. Pacing Clin Electrophysiol. 2018;41:182–193. 29266438 10.1111/pace.13260

[8] Liu CF , Ip JS , Lin AC , Lerman BB . Mechanistic heterogeneity of junctional ectopic tachycardia in adults. Pacing Clin Electrophysiol. 2013;36:e7–e10. 21954877 10.1111/j.1540-8159.2011.03214.x

[9] Dubin AM , Cuneo B , Strasbrger J , Wakai RT , Van Hare GF . Congenital junctional tachycardia and congenital complete AV block: a shared aetiology? Heart rhythm. 2005;2:313–315. 15851326 10.1016/j.hrthm.2004.11.016

[10] Saul JP . Special consideration for ablation in paediatric patients In: Huang SKS, Miller JM, editors. Catheter ablation of cardiac arrhythmias, 4th edn Philadelphia, PA: Elsevier, 2020; p. 664–687.

[11] Cunningham MEA , Doroshow R , Olivieri L , Moak JP . Junctional ectopic tachycardia secondary to myocarditis associated with sudden cardiac arrest. Heart Rhythm Case Rep. 2017;3:124–128. 10.1016/j.hrcr.2016.09.015 PMC5420052 28491785

[12] Namboodiri N , Bohora S , Ajttkumar VK , Tharkan JA . Narrow complex tachycardia with ventriculoatrial dissociation—what is the mechanism? Pacing Clin Electrophysiol. 2011;34:756–759. 21649669 10.1111/j.1540-8159.2010.03001.x

[13] Biase LD , Gianni C , Bagliani G , Padeletti L . Arrhythmias involving the atrioventricular junction. Card Electrophysiol Clin. 2017;9:435–452. 28838549 10.1016/j.ccep.2017.05.004

[14] Ho RT , Pietrasik G , Greenspon AJ . A narrow complex tachycardia with intermittent atrioventricular dissociation: what is the mechanism? Heart Rhythm. 2014;11:2116–2119. 24973561 10.1016/j.hrthm.2014.06.028

[15] Correa FS , Lokhandwala Y , Sánchez‐Quintana D , Mori S , Anderson RH , Wellens HJJ , Sternick EB . Unusual variants of pre‐excitation: from anatomy to ablation: part III—clinical presentation, electrophysiologic characteristics, when and how to ablate nodoventricular, nodofascicular, fasciculoventricular pathways, along with considerations of permanent junctional reciprocating tachycardia. J Cardiovasc Electrophysiol. 2019;30(12):3097–3115. 31646696 10.1111/jce.14247

[16] Han FT . Incessant palpitations and narrow complex tachycardia. Card Electrophysiol Clin. 2016;8:61–65. 26920171 10.1016/j.ccep.2015.10.005

[17] Lau EW . Infraatrial supraventricular tachycardias: mechanisms, diagnosis, and management. Pacing Clin Electrophysiol. 2008;31:490–498. 18373770 10.1111/j.1540-8159.2008.01020.x

[18] Singh DK , Viswanathan MN , Tanel RE , Lee RJ , Lee BK , Marcus GM , Olgin JE , Han F , Vedantham V , Tseng ZH , Pellegrini C , Kawamura M , Gerstenfeld EP , Badhwar N , Scheinman M . His overdrive pacing during supraventricular tachycardia: a novel manoeuvre for distinguishing atrioventricular nodal reentrant tachycardia from atrioventricular reciprocating tachycardia. Heart Rhythm. 2014;11:1327–1335. 24793458 10.1016/j.hrthm.2014.04.038

[19] Bassiounty M , Kanj MH , Tchou P . Ablation of atriofascicular accessory pathways and variants In: Huang SKS, Miller JM, eds. Catheter ablation of cardiac arrhythmias, 4th edn. Philadelphia, PA: Elsevier, 2020; p. 409–428.

[20] Han FT , Riles EM , Badhwar N , Scheinman MM . Clinical features and sites of ablation for patients with incessant supraventricular tachycardia from concealed nodofascicular and nodoventricular tachycardias. J Am Coll Cardiol EP. 2017;3:1547–56. 10.1016/j.jacep.2017.07.015 29759837

[21] Chen H , Shehata M , Cingolani E , Chugh SS , Chen M , Wang X . Differentiating atrioventricular nodal re‐entrant tachycardia from junctional tachycardia conflicting reponses? Circ Arrhythm Electrophysiol. 2015;8:232–235. 25691557 10.1161/CIRCEP.114.002169

[22] Veenhuyzen GD , Quinn FR . Principles of entrainment: diagnostic utility for supraventricular tachycardia. Indian Pacing Electrophysiol J. 2008;8(1):51–65. 18270602 PMC2231607

[23] Miller JM , Das MK , Zipes DP . Case studies in clinical cardiac electrophysiogy, 1st edn Philadelphia, PA: Elsevier, 2018; p. 211–24.

[24] Padanilam BJ , Manfredi JA , Steinberg LA , Olson JA , Fogel RI , Prystowsky EN . Differentiating junctional tachycardia and atrioventricular node re‐entry tachycardia based on response to atrial extrastimulus pacing. J Am Coll Cardiol. 2008;52:1711–7. 19007691 10.1016/j.jacc.2008.08.030

[25] Fan R , Tardos JG , Almasry I , Barbera S , Rashba EJ , Iwai S . Novel use of atrial overdrive pacing to rapidly differentiate junctional tachycardia from atrioventricular nodal reentrant tachycardia. Heart Rhythm. 2011;8:840–844. 21220046 10.1016/j.hrthm.2011.01.011

[26] Hamdan HH , Kalman JM , Lesh MD , Lee RJ , Saxon LA , Dorostkar P , Scheinman MM . Narrow complex tachycardia with VA block: diagnostic and therapeutic implications. Pacing Clin Electrophysiol. 1998;21:1196–1206. 9633061 10.1111/j.1540-8159.1998.tb00178.x

[27] Valdés SO , Landstrom AP , Schneider AE , Miyake CY , de la Uz CM , Kim JJ . Intravenous sotalol for the management of postoperative junctional ectopic tachycardia. Heart Rhythm Case Rep. 2018;4:375–377. 10.1016/j.hrcr.2018.05.007 PMC6092634 30116712

[28] Yoneyama F , Tokunaga C , Kato H , Nakajima T , Mathis BJ , Sakamoto H , Hiramatsu Y . Landiolol hydrochloride rapidly controls junctional ectopic tachycardia after pediatric heart surgery. Pediatr Crit Care Med. 2018;19(8):713–717. 29677032 10.1097/PCC.0000000000001573

[29] Aoki H , Suzuki F , Matsui H , Yasukochi S , Saiki H , Senzaki H , Nakamura Y . Efficacy of a pure Ikr blockade with nifekalant in refractory neonatal congenital junctional ectopic tachycardia and careful attention to damaging the atrioventricular conduction during the radiofrequency catheter ablation in infancy. Heart Rhythm Case Rep. 2017;3:298–301. 10.1016/j.hrcr.2017.03.005 PMC5469282 28649501

[30] He D , Sznycer‐Taub N , Cheng Y , McCarter R , Jonas RA , Hanumanthaiah S , Moak JP . Magnesium lowers the incidence of postoperative junctional ectopic tachycardia in congenital heart surgical patients: is there a relationship to surgical procedure complexity? Pediatr Cardiol. 2015;36(6):1179–1185. 25762470 10.1007/s00246-015-1141-5 PMC4561858

[31] Brochu BD , Abdi‐Ali A , Shaw J , Quinn FR . Successful radiofrequency ablation of junctional ectopic tachycardia in an adult patient. Heart Rhythm Case Rep. 2018;4:251–255. 10.1016/j.hrcr.2018.03.002 PMC6006492 29922584

[32] Tawseef Dar T , Turagam MK , Yarlagadda B , Parikh V , Pillarisetti J , Gopinathannair R , Gianni C , Mohanty S , Mansour M , Di Biase L , Bunch TJ , Natale A , Lakkireddy D . Outcomes of junctional ectopic tachycardia ablation in adult population—a multicenter experience. J Interv Cardiac Electrophysiol. 2020 10.1007/s10840-020-00749-3 32451798

